# The Attitude of Great Writers Towards Disease

**Published:** 1892-12-17

**Authors:** 


### The Attitude of Great Writers Towards Disease.

### XIX.—CHARLES LAMB.

Had Charles Lamb an attitude towards anything? Hardly. But "sworn brother to adversity" from early life, he had a method peculiarly his own of dealing with suffering, mental or physical. He played with it, teased it, stabbed it with gay pricks of satire, tore from it all shadow of disguise, and took it to his inner life with a high endurance of which he seems to have been entirely unconscious. We cannot here do more than touch on the sane tone which he adopted towards his own early attack of insanity, nor on the tender skill with which he shielded his sister during the gradually shortening intervals between her recurrent attacks. Of this never-ending grapple at close quarters with a deadly enemy there is small trace in his writings, only simple references as to a well-known facts in letter to familiar friends. We turn to his mock skirmishes with his own and his friends' pains.

He confessed himself not easily moved by the sight of suffering, and being once rather amused by the contortions of a man with the gout, he reflects: "What is the reason we do not sympathise with pain, short of some terrible surgical operation? Hazlitt, who boldly says all he feels, avows that not only he does not pity sick people, but he hates them. I obscurely recognise his meaning. Pain is probably too selfish a consideration, too simply a consideration of self-attention."

But what enchanting consolations he could offer to his sick friends! What bile could have withstood the generous action of such pleadings as these addressed to Bernard Barton when frightened at his doctor's warnings against his sedentary mode of life? "You are too much apprehensive of your complaint. The best way in these cases is to keep yourself as ignorant as you can, as ignorant as the world was before Galen, of the entire inner construction of the animal man; not to be conscious of a midriff; to hold kidneys (save of sheep and swine) to be an agreeable fiction; not to know where about the gall grows; to acknowledge no mechanism not visible. For once fix the seat of your disorder and your fancies flux into it like bad humours. Above all use exercise, take a little more spirituous liquors, learn to smoke, continue to keep a good conscience, and avoid tampering with hard terms of art—viscosity, scirrhosity, and those bug-bears by which simple patients are scared into their graves. It is the mind, good B. B., and not the limbs that taints by long sitting. Think of the patience of tailors! Think how long the Lord Chancellor sits! Think of the brooding hen!" That last argument must have been irresistible.

What a delightful fiction was this, too, with which he entertained himself and his rheumatic friend. He writes first, "I have these three days been laid up with strong rheumatic pains, in loins, back, shoulders. I shriek sometimes from the violence of them. I get scarce any sleep." Here follows a list of symptoms, ending, "I am ashamed to whine about these complaints to you, who can ill enter into them; but, indeed, they are sharp. You go about in rain or fine, at all hours, without discommodity. I envy you your immunity at a time of life not much removed from my own. But you owe your exemption to temperance, which it is too late for me to pursue. Hence my frame is brittle, yours strong as brass," and more to the same purpose. A week later he writes, "I do confess to mischief. It was the subtlest, diabolical piece of malice heart of man has contrived. I have no more rheumatism than that poker. Never was freer from all aches and pains. The report of thy torments was blown circuitously here from Bury. I could not resist the jeer. I conceived you writhing when you should just receive my congratulations. How mad you'd be! Well, it is not my method to inflict pangs, I leave that to Heaven; but in the existing pangs of a friend I have a share. His disquietude crowns my exemption." But the letter, ending, "Your affectionate and truly healthy friend," should be read in full.

He had a wonderful knack of hitting off small ailments with a graphic touch or two. How many influenza patients have tried ineffectually to explain that they felt like this? "Do you know what it is to succumb under an insurmountable day-mare; an indisposition to do anything or be anything; a total deadness and distaste, a suspension of vitality; an indifference to locality; a numb, soporifical, good-for-nothingness; an ossification all over; an oyster-like insensibility to the passing events; a mind stupor; a brawny defiance to the needles of a thrusting-in conscience? Did you ever have a very bad cold, with a total irresolution to submit to water-gruel processes? I have not a thing to say; nothing is of more importance than another; I am flatter than a denial or a pancake. I inhale suffocation; I can't distinguish veal from mutton; nothing interests me. I sleep in a damp room, but it does me no good. I come home late o' nights, but do not find any visible amendment. Who shall deliver me from the body of this death?"

And what a description this is of deafness: "My head has been a ringing chaos, like the day the winds were made, before they submitted to the discipline of a weather-cock, before the quarters were made. I can hardly read a book, for I miss that small, soft voice which the idea of articulated words raises (almost imperceptibly to you) in a silent reader. I seem too deaf to see what I read."

We conclude with the famous passage from the essays on the egotism of the invalid, a happy dissection of a not uncommon yet hardly acknowledged sentiment: "If there be a regal solitude it is a sick bed. How the patient lords it there! what caprices he acts without control! how king-like he sways his pillow—tumbling and tossing and shifting and lowering and thumping and flatting and moulding it, to the ever-varying requisitions of his throbbing temples. How sickness enlarges the dimensions of a man's self to himself! he is his own exclusive object; supreme selfishness is inculcated upon him as his only duty. 'Tis the Two Tables of the Law to him. He has nothing to think of but how to get well. What a world of foreign cares are merged in that absorbing consideration! He has put on the strong armour of sickness, he is wrapped in the callous hide of suffering; he keeps his sympathy like some curious vintage, under trusty lock and key for his own use only. He lies pitying himself, honing and moaning to himself; he yearneth over himself; his bowels are even melted within him to think what he suffers; he is not ashamed to weep over himself." But he passes into the stage of convalescence, and then—

"Farewell with him all that made sickness pompous: the spell that hushed the household, the mute attendance, the inquiry by looks, the still softer delicacies of self-attention, the sole and single eye of distemper alonely fixed upon itself, world thoughts excluded, the man a world unto himself—his own theatre.

"What a speck is he dwindled into!"

